# What should we know about dementia in the 21st Century? A Delphi consensus study

**DOI:** 10.1186/s12877-015-0008-1

**Published:** 2015-02-06

**Authors:** Michael J Annear, Christine Toye, Frances McInerney, Claire Eccleston, Bruce Tranter, Kate-Ellen Elliott, Andrew Robinson

**Affiliations:** 1 Wicking Dementia Research and Education Centre, University of Tasmania, Medical Sciences 1, 17 Liverpool St, Hobart, 7000 Australia; 2 School of Nursing & Midwifery, Curtin University, Western Australia, GPO BOX U1987, Perth, WA 6845 Australia; 3 School of Social Sciences, University of Tasmania, Private Bag 22, Hobart, 7001 Australia; 4 School of Health Sciences, University of Tasmania, Medical Sciences 1, 17 Liverpool St, Hobart, 7000 Australia

**Keywords:** Dementia, Knowledge, Delphi study, Consensus, Quality of life

## Abstract

**Background:**

Escalating numbers of people are experiencing dementia in many countries. With increasing consumer needs, there is anticipated growth in the numbers of people providing diagnostic evaluations, treatments, and care. Ensuring a consistent and contemporary understanding of dementia across all of these groups has become a critical issue. This study aimed to reach consensus among dementia experts from English speaking countries regarding essential and contemporary knowledge about dementia.

**Methods:**

An online Delphi study was conducted to examine expert opinion concerning dementia knowledge with three rounds of data collection. A sample of dementia experts was selected by a panel of Australian experts, including a geriatrician and three professors of aged care. Purposive selection was initially undertaken with the sample expanded through snowballing. Dementia experts (*N* = 19) included geriatricians, psychologists, psychiatrists, neuroscientists, dementia advocates, and nurse academics from the United Kingdom, United States, and Australia. In the first round, these participants provided open-ended responses to questions determining what comprised essential knowledge about dementia. In the second round, responses were summarised into 66 discrete statements that participants rated on the basis of importance. In the third round, a rank-ordered list of the 66 statements and a group median were provided and participants rated the statements again. The degree of consensus regarding importance ratings was determined by assessing median, interquartile range, and proportion of experts scoring above predetermined thresholds. Correlation scores were calculated for each statement after the final round to identify changes in statement scores.

**Results:**

The Delphi experts identified 36 statements about dementia that they considered essential to understanding the condition. Statements about care for a person experiencing dementia and their care giver represented the largest response category. Other statements, for which full or very high consensus was reached, related to dementia characteristics, symptoms and progression, diagnosis and assessment, and treatment and prevention.

**Conclusions:**

These results summarise knowledge of dementia that is considered essential across expert representatives of key stakeholder groups from three countries. This information has implications for the delivery of care to people with the condition and the development of dementia education programs.

## Background

The prevalence of dementia is increasing worldwide commensurate with population ageing, yet treatments are generally ineffective at slowing progression [1,2]. Evidence increasingly supports the terminal course of dementia and requirements for higher levels of care in the later stages of the condition [3]. People experiencing dementia are encountered in the community, residential aged care facilities (nursing homes), and hospitals. Due to the growing prevalence of people with the condition in the community, the need for knowledge about dementia has never been greater. Knowledge about dementia across different community groups has been found to vary. This includes differences associated with gender [4] and between cohorts of individuals who provide paid and unpaid care [5]. It is possible that such differences in knowledge may be moderated by stigma of the condition, and over one-third of the general population have been reported to hold stereotypical or discriminatory views about dementia [6]. Knowledge about dementia can arguably enhance appropriate diagnosis, prognosis, therapeutic strategies; maintenance of independence, dignity, and safety; and care, including at the end of life [7]. Knowledge is also important to inform social inclusion and psychosocial support for people experiencing dementia and those providing care, including family members [7]. It is essential, therefore, that a systematic approach to dementia knowledge is developed to ensure an objective and up-to-date understanding of dementia among care providers and the general public.

In recognition of its bourgeoning prevalence, researchers have attempted to measure dementia knowledge among cohorts of individuals who provide care. Target populations of dementia knowledge research reported in the international literature include general practitioners [8], public health service workers [9], aged care facility staff [10], and family members of people who have the condition [5]. Among these cohorts, researchers have measured aspects of knowledge that relate to biomedical and clinical components of dementia, including pathology, diagnosis, behaviours and symptoms, risk factors, prevalence, and treatments [8,10]. Research addressing care and management of the person with dementia, including palliative and person-centred approaches, is comparatively uncommon, which is surprising considering growing evidence of the terminal nature of the condition [2].

Four studies undertaken during the last decade have identified dementia knowledge deficits among health workers and family members. A randomized controlled trial involving 127 general practitioners in the United Kingdom (UK) reported that clinicians had an acceptably high knowledge of dementia, but a comparatively poor awareness of the epidemiology of the condition and best-practice diagnostic strategies [8]. Research involving 360 Australian health workers (medical, nursing, and support staff) identified that knowledge of dementia was closely associated with level of education, with medical staff scoring higher than nurses and support staff posting the lowest scores [9]. Areas where most health workers had particularly poor knowledge included the correct identification of risk factors and an understanding of the clinical course of the disease [9]. A further study of 254 aged care staff in the UK showed that this cohort had only a moderate level of knowledge about dementia, which was correlated with lower educational attainment and professional training [10]. Many care workers in the study could not correctly differentiate the symptoms of dementia from normal ageing, while identifying improbable symptoms (headaches and joint pain) as manifestations of the condition [10]. Finally, a recently published Australian study (undertaken by Wicking Dementia Research and Education Centre) concerning knowledge of dementia among 279 aged care staff and 164 family members of people with the condition identified discernable deficiencies [5]. In line with the studies described above, more educated nursing staff exhibited greater levels of dementia knowledge than care workers and family members, although there was wide variation in knowledge among all groups. All groups exhibited poor levels of knowledge on items measuring understanding about the terminal course of dementia and physical symptoms [5]. These studies reveal that dementia knowledge deficits exist among a range of health professionals and family caregivers across several domains. They also suggest that higher levels of education help to mitigate poor knowledge.

Given the known risks associated with dementia, such as an increased risk of falling and respiratory problems, the condition may contribute to a health crisis in the home when strategies to minimize such risks are not addressed. In addition, there may be a failure to meet the complete needs of a person with a terminal illness [8]. Emerging evidence for the terminal course of dementia [3] has particular implications for those who provide both medical and daily care. For example, failure to correctly diagnose and manage a terminal illness can have significant and deleterious impacts on quality of life for a person with the condition. This can include invasive, burdensome, and futile interventions and procedures near the end of life [3]. Considering the complexity of dementia as a multi-faceted syndrome with diverse underlying pathologies, its terminal nature, and related knowledge deficiencies among health workers and family members, it is critical to establish essential aspects of knowledge about dementia that can support care.

This study employed a Delphi method to reach consensus among experts from English-speaking countries regarding knowledge that they considered important for understanding dementia. Experts were asked to provide factual information about dementia that they considered important for the provision of care and treatment for people with the condition that would be relevant for both health professionals and lay people. This was important as the accelerating global growth of dementia [1] will not only challenge the public health system, but will also see greater numbers of people with neurodegenerative conditions ageing in place in communities of their choice [11]. In this context, families and members of the general public will experience increasing encounters with people with dementia in the years ahead. The Delphi technique was originally conceived by researchers at the RAND Corporation in the 1950s as a method for achieving a convergence of opinion on real-world knowledge among experts within prescribed topic areas [12,13]. A small number of Delphi studies have previously been employed by researchers to build consensus in the areas of dementia prevalence in developing countries [1], standard definitions of dementia palliative care [14], and essential components of case management for people with dementia who live in the community [15]. While these studies have sought a convergence of expert opinion in relation to the scale, definition, and care approaches for dementia, we are not aware of any studies that have used this method to identify contemporary knowledge of dementia care that is arguably central to driving evidence-based practice, education, and policy.

## Methods

### Design

The main components of Delphi studies are the systematic canvassing of expert opinion over a series of rounds using open and closed ended questions, ensuring participant anonymity to reduce the potential for dominant individuals to influence group opinion, and statistical investigation of group responses [12]. Delphi studies are most commonly undertaken over two to four feedback rounds with samples of between 10 and 20 expert respondents [12], although there is little consensus in the literature concerning optimal sample size [16]. The number of rounds considered sufficient in Delphi studies varies, although more rounds increases potential response fatigue and participant withdrawal [17], which can be problematic in studies with smaller sample sizes. In this Delphi study, three feedback rounds were employed using an online web form hosted on a secure University server in order to balance convenience of engagement with the time constraints of busy clinicians and researchers. Three phases were employed in the study: 1) identifying important information about dementia; 2) rating knowledge statements; and 3) confirming group consensus.

### Participants

In Delphi studies, participant selection is often regarded as the most important step in the process because it relates directly to the quality of the data generated [12,18]. Participant selection relies on a consideration of disciplinary area, target issue, and level of training or experience [18]. The judgment and discretion of the researchers is ultimately tested in the selection of potential participants and, for this reason, a team of experienced researchers often nominate potential participants [16]. A panel of researchers and clinicians with expertise in dementia identified a pool of Australian and international experts. The panel included professors of aged care nursing (AR, FM, CT), a geriatrician (SM), a gerontologist (MA), and a clinical psychologist (KE). Potential experts were identified in the areas of clinical care, dementia education, advocacy, service delivery, psychiatry, clinical psychology and clinical neuropsychology. Snowball sampling was also used. Potential respondents were asked to nominate other experts who they felt might be able to contribute to the study. The research was reviewed and approved by the University of Tasmania Human Research Ethics Committee (H0013532). Study participants remained anonymous to each other throughout the research process and each respondent was assigned a unique alpha-numeric code to allow them to access the online Delphi information and to ensure that their confidentiality was maintained throughout the study. Experts were contacted by email directly by the researcher after a referral from members of the expert panel. One follow-up email was sent in each round if experts did not respond within two weeks. If an expert did not respond to the survey after the follow-up email, they were considered to have dropped out of the study.

### Round one (December 2013): generating information

In the first round of the Delphi study, experts were asked to provide open ended answers to questions about important dementia knowledge. The following questions were posed to respondents:

- How would you define dementia for a lay person?
- What key facts are essential to understanding dementia?
- What key facts about dementia are frequently misunderstood by lay people?
- What key facts about dementia should people in your field know?

Respondents were also asked to describe any knowledge areas inadequately addressed in existing measures of dementia knowledge. These questions were posed to elicit factual information about dementia that experts considered to be essential to understanding the condition among individuals with varying education, understanding, or experience. The questions were also used to identify facts about dementia that were not currently well understood among general and expert groups. The questions were generated in discussion with the expert panel described in the previous section.

Two researchers independently reviewed the respondent feedback in this phase and developed a list of statements containing information about dementia based on expert responses. Each researcher read all participant responses and summarized these into the minimum number of statements necessary to accurately reflect all perspectives. Where possible, statements were provided in respondents’ own words to limit researcher bias [19]. Changes were only made to statements to improve clarity of expression. These lists were then integrated into a master list of statements that experts considered to be important for understanding dementia.

### Round two (February 2014): rating knowledge statements

In the second round, respondents were presented with the statements identified in the first round and asked to rate each one in relation to how important it was for understanding dementia on a five-point Likert scale: 1 (not important at all) to 5 (very important). Participants were also asked whether the statements covered all of the important knowledge about dementia and were provided with the option of suggesting additional information. Respondent ratings were then analysed to identify items that had both the highest ratings and highest levels of consensus among the expert group.

### Round three (March 2014): confirming consensus

In the final round of the Delphi process, respondents were provided with a list of statements from round two as well as the group score (median) for each item. Items were listed according to their consensus rank: full consensus, very high consensus, high consensus, moderate consensus, or low consensus – an approach previously used in other published studies [14]. Full and very high consensus indicated that experts regarded a particular item as essential for understanding dementia. Respondents were asked to review the item’s median score and rank and to then, in congruence with the second round, assign a score from 1 (not important at all) to 5 (very important) to each item. Scores from the final round were then tallied to identify the final high consensus items. This is a useful consideration as changes in item scores between Delphi rounds are an indication of the stability of consensus.

### Measurement and analysis

Consensus within Delphi studies is typically defined by the percentage of responses falling within a prescribed range. For example, some researchers have previously recommended a benchmark of 75–80% of respondents falling within a particular range of scores as an indication of acceptable consensus [17,20]. Common statistics used in Delphi studies include measures of central tendency (such as a median) and level of dispersion (such as inter-quartile range) [19]. Researchers have also suggested that an appropriate measure is also the stability of consensus across Delphi rounds, which indicates whether consensus has stayed the same, increased, or decreased [20]. The scoring system used in this study was adapted from research published by Van Der Steen and colleagues [14], and structured to identify statements of both high importance and high consensus. Full consensus was considered to be a median score of 5, an interquartile range of 0, and 100% of respondents rating the statement with the highest possible score (5). Very high consensus was considered to be a median score of 5, an interquartile range of 0, and ≥80% of experts scoring the item either a 4 or a 5. Finally, the non-parametric Wilcoxon Signed Rank Test was employed to ascertain whether there was a significant difference in individual item scores between rounds 2 and 3.

## Results

The research panel identified 35 experts (including experts from the United Kingdom, the United States, France, Singapore, Malaysia, and Hong Kong) who were invited to be involved in the research. Respondents nominated a further three experts who were also invited to participate in the study. Of the 38 experts who were approached to participate in the study, 19 participants (50% response rate) from three countries completed the first round of the Delphi study. Two participants dropped out between rounds one and two and a further two participants dropped out between rounds two and three (an overall attrition rate of 21% over three rounds). Reasons provided for refusal or drop out included time commitments and periods of planned or unscheduled leave that interrupted continuity of the research process. Most responders were from Australia (*n* = 15 during round one), with smaller numbers from the United States (*n* = 2) and United Kingdom (*n* = 2). No responses were received from respondents in France, Malaysia, Singapore, or Hong Kong. Of those who participated in the study, the most frequently acknowledged areas of expertise were clinical care and dementia education (Table 1).

**Table 1:** **Characteristics of Delphi participants**

	Round 1 (n = 19)	Round 2 (n =17)	Round 3 (n = 15)
Female gender	8	7	6
Country			
Australia	15	13	11
United States	2	2	2
United Kingdom	2	2	2
Occupation			
University academic	11	10	9
Clinician (Geriatrician or nurse specialist)	6	5	4
Not for profit/advocacy organization manager	2	2	2
Reported areas of expertise^1^			
Clinical care	35%		
Dementia education	23%		
Advocacy	13%		
Service delivery	13%		
Neuropsychology	10%		
Research	6%		

^1^Participants were free to select more than one area of expertise.

### Round one: identifying factual statements about dementia

In the first round of the study, 19 participants provided statements about dementia that they considered essential to understanding the condition. After receiving expert comments, two researchers independently summarized the open-ended feedback into the minimum number of statements that could reflect the consolidated expert information. In total, 66 statements about dementia were identified from expert feedback. Expert statements were grouped into thematic categories by two researchers (MA and CE) for the purposes of data organization and analysis. Thematic categories included basic characteristics of dementia, symptoms and progression, diagnosis and assessment, treatment and prevention, and care for people with dementia. The accuracy of identified categories was confirmed by a geriatrician (JT) with 15 years of experience in dementia care (Table 2).

**Table 2:** **Expert statements considered important for a contemporary understanding dementia (*N*** **= 66)**

**A:**	**Basic characteristics of dementia (14% of total items)**
1	Dementia refers to a group of diseases that affect the brain.
2	People with dementia have a disease that affects the structure and function of their brain.
3	Dementia is not a psychological condition.
4	Dementia always becomes worse over time.
5	Dementia is a terminal condition that will result in death.
6	Dementia most commonly affects older adults.
7	Dementia can occur in younger adults.
8	Dementia is not a part of normal ageing.
9	Prevalence of dementia is increasing within many societies.
**B:**	**Symptoms and progression (30% of total items)**
10	Dementia affects people across five domains: cognitive, functional, psychiatric, behavioural, and physical.
11	Difficulty swallowing is a symptom of dementia.
12	Difficulty with movement is a symptom of dementia.
13	Difficulty speaking is a symptom of dementia.
14	Memory loss is a symptom of dementia.
15	Behavioural changes are symptoms of dementia.
16	Personality changes are symptoms of dementia.
17	Wandering is a symptom of dementia.
18	Confusion is a symptom of dementia.
19	Difficulty making decisions is a symptom of dementia.
20	Difficulty with problem solving is a symptom of dementia.
21	Difficulty with learning is a symptom of dementia.
22	Cognitive and functional losses can fluctuate in a person with dementia.
23	Symptoms of dementia differ by type of underlying disease.
24	People with dementia often have other chronic medical conditions.
25	It may take years for dementia to develop to the point that it affects cognition and functioning.
26	Dementia has discernable stages.
27	The course of dementia is unpredictable.
28	It is possible to communicate with a person who has advanced dementia.
29	A person with advanced dementia may be able to communicate non-verbally.
**C:**	**Diagnosis and assessment (17% of total items)**
30	Alzheimer’s disease is the most common form of dementia.
31	Dementia with Lewy Bodies is a common form of dementia.
32	Vascular dementia is a common form of dementia.
33	Fronto-temporal dementia is a common form of dementia.
34	Definitive diagnosis of dementia in a living patient is difficult.
35	Early diagnosis of dementia improves treatment outcomes.
36	A high proportion of people who have dementia do not have a diagnosis.
37	Assessment of a person with dementia is important to determine whether they are suffering from treatable and co-existing medical and psychiatric conditions.
38	Depression in a person with dementia should be identified and treated.
39	Delirium should be ruled out in a person with suspected dementia.
40	Pain in a person with dementia should be identified and treated.
**D:**	**Treatment and prevention (15% of total items)**
41	There is currently no cure for dementia.
42	Symptoms of dementia can be improved with medication.
43	Psychosocial interventions can improve quality of life for people with dementia.
44	Non-pharmacological interventions are often more appropriate for treating dementia-related behavioural problems.
45	Psychotropic medications may cause undue harm to a person with dementia.
46	Cognitive stimulation for the person with dementia can improve symptoms.
47	Exercise for the person with dementia can improve symptoms.
48	Some of the risk factors for dementia are modifiable.
49	A healthful lifestyle can reduce the risk of developing dementia.
50	In most cases, having parents with dementia does not greatly increase the risk of developing the condition.
**E:**	**Care for people with dementia (24% of total items)**
51	It is possible for a person with dementia to live independently during the initial stages of the condition.
52	Most people who have dementia live in their own homes in the community.
53	People with dementia are not always a risk to themselves and others.
54	People with dementia will eventually require a high level of care and assistance with activities of daily living.
55	It is important to plan the future care of a person once a diagnosis of dementia has been made.
56	Education following diagnosis is important to help a person with dementia and their carer to manage the condition.
57	The wishes of a person with dementia should be taken into account when planning for their treatment and care.
58	A palliative approach to care is appropriate for a person with dementia.
59	A person-centred approach to care is appropriate for a person with dementia.
60	Aggressive and invasive treatments are often not appropriate for people with dementia.
61	Relationships remain important for a person with dementia.
62	People with dementia should continue to be involved in meaningful physical, social, and mental activities.
63	Caring for a person with dementia can be stressful.
64	Caregivers of people with dementia require support.
65	A person with dementia may retain more understanding than they can express.
66	A person’s past can be important for understanding behavioural problems.

### Round two: rating knowledge statements

In the second round of the Delphi study, 17 remaining experts rated each of the 66 statements about dementia on their perceived importance on an ordinal scale running from 1 (not important at all) to 5 (very important). Two participants did not provide feedback after a follow-up email was sent to all respondents from the first round. In addition to rating statements, participants were also provided with an option to suggest statements that had not been included in the list or to comment on the research process. Six experts made comments about the wording and language used in the statements, although no new content suggestions were made.

### Round three: confirming consensus

In the final round of the Delphi study, the 15 remaining experts rated each of the 66 statements a second time. Two participants did not provide feedback after a follow-up email was sent to all respondents from the second round. Full consensus was achieved for 5 of the 66 items in the final round. Very high consensus was achieved for 31 of the 66 items. Statistically significant increases in statement rating between rounds two and three were observed for the following four statements (see Table 3): 1) assessment of a person with dementia is important to determine whether they are suffering from treatable and co-existing medical and psychiatric conditions (very high to full consensus); 2) aggressive and invasive treatments are often not appropriate for people with dementia (high to very high consensus); 3) caring for a person with dementia can be stressful (high to very high consensus); and 4) a person with dementia may retain more understanding than they can express (high to very high consensus).

**Table 3:** **Delphi consensus statements**

Item	Category	Interquartile range	Responders scoring 5 in round 3 (%)	Score change between rounds 2 and 3 (***z***)	Significance (***p***)
**Full consensus items (*n*** **= 5)**					
Dementia refers to a group of diseases that affect the brain	Characteristics of dementia	0	100%	-1.63	.10
Behavioural changes are symptoms of dementia	Symptoms and progression	0	100%	-2.00	.05
Assessment of a person with dementia is important to determine whether they are suffering from treatable and co-existing medical and psychiatric conditions	Diagnosis and assessment	0	100%	-2.00	.04*
Non-pharmacological interventions are often more appropriate for treating dementia-related behavioural problems	Treatment and prevention	0	100%	-1.63	.10
It is possible for a person with dementia to live independently during the initial stages of the condition.	Care for people with dementia	0	100%	-1.89	.06
**Very high consensus items (*n*** **= 31)**					
People with dementia have a disease that affects the structure and function of their brain.	Characteristics of dementia	0	93%	-1.51	.13
Dementia is not a part of normal ageing.	Characteristics of dementia	0	87%	-1.13	.26
Dementia is a terminal condition that will result in death.	Characteristics of dementia	0	80%	-1.47	.14
Dementia most commonly affects older adults.	Characteristics of dementia	0	80%	-2.00	.05
Memory loss is a symptom of dementia.	Symptoms and progression	0	93%	-1.63	.10
Difficulty with problem solving is a symptom of dementia.	Symptoms and progression	0	93%	-1.89	.06
Dementia affects people across five domains: cognitive, functional, psychiatric, behavioural, and physical.	Symptoms and progression	0	87%	-1.47	.14
Difficulty making decisions is a symptom of dementia.	Symptoms and progression	0	87%	-1.41	.16
Difficulty with learning is a symptom of dementia.	Symptoms and progression	0	87%	-1.73	.08
It is possible to communicate with a person who has advanced dementia.	Symptoms and progression	0	87%	-1.41	.16
A person with advanced dementia may be able to communicate non-verbally.	Symptoms and progression	0	87%	-1.41	.16
Pain in a person with dementia should be identified and treated.	Diagnosis and assessment	0	93%	-1.41	.16
Delirium should be ruled out in a person with suspected dementia.	Diagnosis and assessment	0	87%	-1.52	.13
Depression in a person with dementia should be identified and treated.	Diagnosis and assessment	0	87%	-1.63	.10
Alzheimer’s disease is the most common form of dementia.	Diagnosis and assessment	0	80%	-1.19	.23
Psychotropic medications may cause undue harm to a person with dementia.	Treatment and prevention	0	93%	-1.86	.06
There is currently no cure for dementia.	Treatment and prevention	0	87%	-1.47	.14
Psychosocial interventions can improve quality of life for people with dementia.	Treatment and prevention	0	80%	-1.27	.21
Some of the risk factors for dementia are modifiable.	Treatment and prevention	0	80%	-1.89	.06
The wishes of a person with dementia should be taken into account when planning for their treatment and care.	Care for people with dementia	0	93%	0.00	1.00
A person-centred approach to care is appropriate for a person with dementia.	Care for people with dementia	0	93%	-1.00	.32
Caring for a person with dementia can be stressful.	Care for people with dementia	0	93%	-2.06	.04*
Caregivers of people with dementia require support.	Care for people with dementia	0	93%	0.00	1.00
A person with dementia may retain more understanding than they can express.	Care for people with dementia	0	93%	-2.24	.03*
Most people who have dementia live in their own homes in the community.	Care for people with dementia	0	87%	-.82	.41
Education following diagnosis is important to help a person with dementia and their carer to manage the condition.	Care for people with dementia	0	87%	-.82	.41
Aggressive and invasive treatments are often not appropriate for people with dementia.	Care for people with dementia	0	87%	-2.06	.04*
Relationships remain important for a person with dementia.	Care for people with dementia	0	87%	0.00	1.00
People with dementia should continue to be involved in meaningful physical, social, and mental activities.	Care for people with dementia	0	87%	-1.41	.16
A person’s past can be important for understanding behavioural problems.	Care for people with dementia	0	87%	-.54	.60
It is important to plan the future care of a person once a diagnosis of dementia has been made.	Care for people with dementia	0	80%	-1.60	.11
**Very low consensus items (*n*** **= 11)**					
Dementia is not a psychological condition.	Characteristics of dementia	2	13%	-.42	.67
Difficulty with movement is a symptom of dementia.	Symptoms and progression	2	27%	-1.30	.19
Difficulty with swallowing is a symptom of dementia.	Symptoms and progression	2	27%	-1.66	.10
People with dementia often have other chronic medical conditions.	Symptoms and progression	2	27%	-.09	.93
The course of dementia is unpredictable.	Symptoms and progression	2	20%	.00	1.00
Wandering is a symptom of dementia.	Symptoms and progression	2	13%	-1.04	.30
Difficulty speaking is a symptom of dementia.	Symptoms and progression	2	13%	-.42	.68
Dementia has discernable stages.	Symptoms and progression	2	6%	-1.31	.19
Early diagnosis of dementia improves treatment outcomes.	Diagnosis and assessment.	2	27%	-1.29	.20
Exercise for the person with dementia can improve symptoms.	Treatment and prevention	2	27%	-1.75	.08
A palliative approach to care is appropriate for a person with dementia.	Care for people with dementia.	2	40%	-.88	.38

All statements had a Median score of 5 and an interquartile range of 0.

*Significant at p < .05.

Statements from all thematic categories were represented in expert assessments of the most important items for understanding dementia. The dominant thematic category for responses was ‘care for people with dementia’ (32% of items identified by experts as highly important for understanding dementia and comprising 24% of total statements). The ‘symptoms and progression’ category contributed the second greatest number of items, although the number of items as a proportion of the total was reduced (22% of items identified by experts as highly important for understanding dementia and 30% of total statements). Lower levels of support were identified for statement categories relating to characteristics of dementia, diagnosis and assessment, and treatment and prevention.

## Discussion

This Delphi study identified 36 (out of 66) statements about dementia that a group of experts consider to be essential for understanding the condition. Within this group of statements, full consensus (100% agreement) was achieved for 5 statements and a very high level of consensus (≥80% agreement) was obtained for 31 statements. There was broad agreement across the group of experts that a contemporary understanding of dementia requires a full consideration of basic characteristics of the syndrome, symptoms and progression, diagnosis and assessment, treatment and prevention, and care for people with dementia. Only four out of 36 of the statements showed a significant change between the second and third Delphi rounds, which is indicative of stability in expert sentiment [20].

### Characteristics of dementia

The expert group agreed that dementia is a terminal syndrome characterized by deterioration in the structure and function of the brain. They considered dementia to be predominantly a condition of later life, but not a part of the normal course of ageing. There is emerging evidence that dementia has a terminal phase and that it leads to mortality through effects on respiratory function (particularly pneumonia), swallowing and eating problems, and febrile episodes [3]. The expert group were united in their consideration that it is appropriate to consider dementia as a terminal condition underpinned by a progressive pathology. The literature indicates that dementias are a deviation from the normal course of aging [21] as conditions with an underlying physical disease process [22]. An understanding of the pathological and terminal nature of dementia is crucial as it allows for timely planning of care and treatment wishes of the person with dementia. Further, the development of person-centred management and treatment plans following timely diagnosis, and preparations for accommodating expected changes in health and functioning, are informed by knowledge about dementia and its trajectory of decline among those providing care.

### Symptoms and progression

The expert group agreed that dementia affects people across multiple domains. Important symptoms that should be recognized include behavioural changes, memory impairments, and difficulties with executive functioning. The expert group agreed that communication with a person with dementia is possible even in later stages of the condition. The international literature has established an evidence base for the symptoms of dementia across a spectrum of cognitive, functional, behavioural, psychiatric, and physical domains that interrupt daily life of a person with dementia [23]. Recognition of symptoms of the condition is important for timely diagnosis and an assessment of the underlying pathology, which may affect treatment and management of the condition. The focus on communication and the capacity for people with dementia to retain the ability to comprehend and interact (including non-verbal communication) is supported in the literature [24] and reflects experts’ views that communication, in varying degrees and types, is possible (though often difficult) even as dementia affects a range of domains and manifests with complex and challenging symptoms. The capacity for continued communication has important implications for the provision of person-centred care and the maintenance of close relationships in the later stages of the condition.

### Diagnosis and assessment

With regards to diagnosis and treatment, the expert group regarded prevalent typologies and co-existing conditions to be important for understanding the condition. The expert group agreed that Alzheimer’s disease is currently the most prevalent form of dementia. They also contended that a person with dementia should be assessed to determine whether they are experiencing co-existing and treatable physical and psychiatric symptoms, including depression, pain, and delirium. Alzheimer’s disease is acknowledged as the most common form of dementia internationally, accounting for more than two thirds of diagnosed cases [25,26]. It is important for caregivers and health professionals to recognize the prevalence of Alzheimer’s disease in the context of their professional practice and amongst the spectrum of diseases that cause dementia in order to understand symptomatology and provide more effective treatment and care that is aligned with the expected course of the condition. People with dementia can have a reduced quality of life resulting from factors including unrecognized pain [27], depression [28], and delirium [29]. If a person with dementia cannot express themselves in a way that is comprehensible to their care giver, or if a care giver is not attuned to the signs and symptoms of co-existing and treatable medical and psychological conditions, then reduced quality of life may result. An understanding of the potential consequences of co-existing conditions and the prevalence of Alzheimer’s disease may prompt increased vigilance among carers and health professionals and result in better care for people experiencing dementia.

### Treatment and prevention

The expert group agreed that there is no cure for dementia and that psycho-social (non-pharmacological) interventions are often most appropriate both to provide quality of life and ameliorate behavioural and psychological symptoms of dementia (as differentiated from symptoms due to pain or agitation at not being understood by care givers). Some of the risk factors for dementia were identified as modifiable in earlier stages of life. Congruent with expert feedback regarding the characteristics of dementia, research evidence supports the position that dementia is an incurable and progressively degenerative condition [3]. Evidence is mixed, however, for the efficacy of pharmaceutical interventions in the management of dementia symptoms. There is evidence of harm (masking communication attempts or side effects attributable to the medicine or interaction with other pharmaceuticals) when a person with dementia is overprescribed psychotropic medication for behavioural and psychological symptoms of dementia (BPSD) [30] that might be better ameliorated by less invasive means [31]. Psychosocial interventions that are person-centred, individually tailored, and non-invasive are often more effective at improving mood, reducing agitation, and addressing depression and anxiety for people with dementia [32,33]. However, pharmaceutical interventions for a person with dementia may be indicated to address pain or infections, which could trigger or exacerbate behavioural and psychological symptoms [34]. In this regard, the use of pharmacological treatments in the person with dementia is complex and requires detailed observation and assessment to correctly ascertain the underlying cause of BPSD. Clearly, the potential for harm in the use of medication should be weighed carefully against the impact on quality of life for people diagnosed with dementia. The expert group suggests that psychosocial interventions require more attention in our 21st Century understanding of dementia.

There is emerging evidence to support the expert view that certain risk factors for dementia may be modifiable in earlier or later life, which may have varying outcomes in relation to the development or progression of dementia. Modifiable risk factors are similar to those associated with cardiovascular disease and certain cancers, including inactivity, poor diet, overweight, low level of education, smoking, and alcohol consumption [35]. Exercise, for example, may reduce risks of developing dementia by limiting the development of diseases of the circulatory system associated with inactivity [36]. Higher levels of education have also been shown to reduce the risk of developing dementia by potentially mitigating the expression of deleterious genetic material that underlies the development of certain dementias [37]. Knowledge about the potential for changing individual risk of developing dementias is important as the transmission of such information could potentially prompt modification of lifestyle factors that contribute to the development or progression of dementia.

### Care for people with dementia

Statements about care were those most frequently identified as essential knowledge by the expert group. Experts considered person-centred strategies to be paramount in the care for persons with dementia. There was consensus that person-centred care provided in the early stages of the condition should focus on supporting independence, planning future care needs based on the wishes of the individual, and educating the person with dementia and their carer. Experts agreed that as the condition progressed, aggressive and invasive treatments should be carefully balanced against the impact on the quality of life for the person with dementia. It was also considered important that efforts be made to continue engagement with loved ones and involvement in meaningful activities.

Person-centred approaches to care (care that is individualized, values based, empathetic, and which provides a supportive social environment) are supported in the literature as having positive impacts on quality of life for people with dementia [38,39]. Within a person-centred framework, education appears to moderate the relationship between care and health outcomes. For example, two randomised trials involving large cohorts of aged care workers and residents have shown that educating staff in person-centred approaches care for people with dementia led to significant improvements in quality of life and reductions in psychological symptoms of dementia and agitation [39,40]. It is apparent that the expert group in this study supports the view that education can promote improved care and quality of life for people with dementia. While many of the respondents reported expertise in dementia education, there is also a growing focus on improving content, quality, context, and delivery in educational endeavours that suggests a greater role for education in dementia care. For example, evidence from online dementia education programs indicates that the outcomes of different initiatives, as measured by factors such as course completion, can be widely diverse and potentially influence efficacy [41].

In relation to quality of life as dementia progresses, harm can result when individuals with the condition are inappropriately administered burdensome medical and pharmacological interventions or transferred into hospital for invasive treatments [30,42]. Experts concurred with this view and, with regard for the terminal course of dementia, supported approaches to care that focus on comfort, quality of life, and meaningful engagement in physical, social, and mental activities. Models of care for people with dementia that focus on maintaining comfort, quality of life, and engagement are preferred by individuals and family members [43,44]. This contention fits with a palliative approach to care (care that improves quality of life for patients and their families facing life-threatening illness) [45] for people with dementia and acknowledges the terminality of the condition. It is notable in this study, however, that the expert group did not reach a very high level of consensus on whether a palliative approach to care was appropriate for a person with dementia. This is perplexing as the group acknowledged that dementia is terminal condition. The lack of consensus concerning the appropriateness of palliative care for people with dementia may be due to negative associations with end of life care, potential for added distress, or perceived loss of hope for the person with the condition and their family members. A systematic review of research into the efficacy of palliative approaches to care in advanced dementia has reported that published studies support the effectiveness of this approach, but that an evidence base is lacking due to prognostic uncertainty among clinicians and a lack of clear outcome measures for patients who are unable to express their needs or wishes [46]. Currently, there is a lack of appropriate tools to estimate survival time for a person with dementia, which arguably hampers clinicians’ ability to effectively ascertain disease progression and associated symptomatology in order to guide treatment and care up to and including end-of-life [47]. A recent Delphi study from Europe has also reached consensus among 63 experts (health professionals and researchers) concerning a standard definition of dementia palliative care [14]. They determined that maximization of comfort for a person with dementia was the highest priority during the progression of the condition, underpinned by person-centred care, communication, shared decision making, advance planning, provision of physical comfort, and treatment of symptoms that reduce quality of life [14]. While the present study did not highlight knowledge of palliative care approaches as essential, a focus on personhood, independence, communication, education, meaning, and quality of life show an alignment with palliative care-related concepts.

### Limitations and strengths of the research

This study sheds light on information that experts consider to be essential for understanding dementia in the 21st century. There is, however, a need to consider potential limitations associated with this study. The majority of respondents in this study were from Australia, with smaller numbers from the United States and the United Kingdom, while the prevailing areas of self-reported expertise within the respondent group were clinical care and dementia education. Potential exists for geographical and demographic biases in this study. The absence of participants from continental Europe, in particular, limits the extent to which these findings can be considered truly representative of the views of an international cohort of experts. This problem arose as a result of the purposive nature of the sampling and the utilization of professional networks among the Australian expert panel that provided contact information for Delphi participants. Comparable international studies have reported similar geographic biases when research involves experts from different countries, particularly where English is a second or third language [48]. While Australian experts dominated the Delphi study sample, global patterns of dementia in more developed countries indicate that experiences of the condition are likely to be similar in the United States, Western Europe, New Zealand, and more developed parts of Asia [1]. For this reason, it is likely that Australian respondents share knowledge about the condition with experts in similarly developed countries. Snowball sampling was also used during the initial recruitment phase in order to provide an opportunity for the involvement of a wider cohort of experts.

Greater numbers of respondents with clinical knowledge of dementia potentially bias the identification and construction of dementia knowledge in this study [49]. It is possible that responses represent a disproportionately medical understanding of the syndrome that reduces or ignores the lived, personal experience [49]. Clinicians and medical researchers may bring different values to studies about appropriate dementia knowledge and, as a result, may overlook legal and ethical issues, end-of-life decision making, and individual human rights [50,51]. It is possible that respondents who have a clinical focus may differ in how they prioritise information about care and treatment for people with dementia when contrasted with, for example, family members or people in other professions (such as law or social work). As an example, researchers from Europe have raised the importance of issues such as continuing participation of people with progressing dementia in social media [50], decision making capabilities in relation to assisted suicide and euthanasia [52], and perceived or ascribed rights of people with dementia at different stages of the condition [53]. Despite higher numbers of respondents who reported clinical expertise, the predominance of statements addressing the importance of care for a person with dementia (embodying notions of personhood, independence, communication, and meaning) suggest that expert viewpoints were relatively nuanced. It is also notable that many experts considered themselves as having expertise in several dementia-related areas, including education, advocacy, and service delivery. Within this limited sample, therefore, a range of perspectives on dementia knowledge were evident.

Another source of potential bias in the data is attributable to the moderate response rate: 50% of identified experts who were approached to participate in the study responded to an invitation. Although a modest response rate was achieved, a relatively low rate of attrition was experienced over three consultation rounds. Delphi studies tend to have low rates of attrition as experts who participate often have an appreciation of research methods and an interest in the study topic [18]. Despite the loss of four respondents between the first and third rounds, the total sample remains within an appropriate range for Delphi studies [12]. Other strengths of the research that reduced bias include the use of a panel of senior academics and clinicians to identify local and international experts and independent extraction of salient qualitative statements in the first Delphi round.

## Conclusion

This study identified information about dementia that experts consider essential for a contemporary understanding of the condition. While expert consensus supports a diverse range of domains as critical in understanding dementia, information about the provision of care was prominent. The findings may assist clinicians and academics to convey information about dementia to colleagues, health workers, and the general public, as well as facilitate the development of education and knowledge-evaluation products.

## References

[1] Ferri CP, Prince M, Brayne C, Brodaty H, Fratiglioni L, Ganguli M (2005). Global prevalence of dementia: a Delphi consensus study. Lancet.

[2] Mitchell SL, Kiely DK, Hamel MB (2004). Dying with advanced dementia in the nursing home. Arch Intern Med.

[3] Mitchell SL, Teno JM, Kiely DK, Shaffer ML, Jones RN, Prigerson HG (2009). The clinical course of advanced dementia. N Engl J Med.

[4] Werner P, Goldberg S, Mandel S, Korczyn AD (2013). Gender differences in lay persons’ beliefs and knowledge about Alzheimer’s disease (AD): a national representative study of Israeli adults. Arch Gerontol Geriatr.

[5] Robinson A, Eccleston C, Annear M, Elliott K, Andrews S, Stirling C (2014). Who knows, Who cares: nurses, care workers and family members’ knowledge of dementia. J Palliat Care.

[6] Blay S, Peluso E (2010). Public stigma: the community’s tolerance of Alzheimer disease. Am J Geriatr Psychiatr.

[7] Arai Y, Arai A, Zarit SH (2008). What do we know about dementia?: A survey on knowledge about dementia in the general public of Japan. Int J Geriatr Psychiatr.

[8] Turner S, Iliffe S, Downs M, Wilcock J, Bryans M, Levin E (2004). General practitioners’ knowledge, confidence and attitudes in the diagnosis and management of dementia. Age Ageing.

[9] Smyth W, Fielding E, Beattie E, Gardner A, Moyle W, Franklin S (2013). A survey-based study of knowledge of Alzheimer’s disease among health care staff. BMC Geriatr.

[10] Hughes J, Bagley H, Reilly S, Burns A, Challis D (2008). Care staff working with people with dementia: training, knowledge and confidence. Dementia.

[11] Brittain K, Corner L, Robinson L, Bond J (2010). Ageing in place and technologies of place: the lived experience of people with dementia in changing social, physical and technological environments. Sociol Health Illness.

[12] Hsu C-C, Sandford BA (2007). The Delphi technique: making sense of consensus. Practical Assess Res Eval.

[13] Dalkey N (1969). The Delphi method: an experimental study of group opinion.

[14] Van Der Steen J, Radbruch L, Hertogh C, de Boer M, Hughes J, Larkin P (2013). White paper defining optimal palliative care in older people with dementia: a Delphi study and recommendations from the European Association for Palliative Care. Palliat Med.

[15] Verkade P-J, van Meijel B, Brink C, van Os-Medendorp H, Koekkoek B, Francke AL (2010). Delphi-research exploring essential components and preconditions for case management in people with dementia. BMC Geriatr.

[16] Akins RB, Tolson H, Cole BR (2005). Stability of response characteristics of a Delphi panel: application of bootstrap data expansion. BMC Med Res Methodol.

[17] Keeney S, Hasson F, McKenna H (2006). Consulting the oracle: ten lessons from using the Delphi technique in nursing research. J Adv Nurs.

[18] Okoli C, Pawlowski SD (2004). The Delphi method as a research tool: an example, design considerations and applications. Inform Manag.

[19] Hasson F, Keeney S, McKenna H (2000). Research guidelines for the Delphi survey technique. J Adv Nurs.

[20] De Meyrick J (2003). The Delphi method and health research. Health Educ.

[21] Frisoni GB, Ganzola R, Canu E, Rüb U, Pizzini FB, Alessandrini F (2008). Mapping local hippocampal changes in Alzheimer’s disease and normal ageing with MRI at 3 Tesla. Brain.

[22] Vemuri P, Simon G, Kantarci K, Whitwell JL, Senjem ML, Przybelski SA (2011). Antemortem differential diagnosis of dementia pathology using structural MRI: differential-STAND. Neuroimage.

[23] Pérès K, Helmer C, Amieva H, Orgogozo JM, Rouch I, Dartigues JF (2008). Natural history of decline in instrumental activities of daily living performance over the 10 years preceding the clinical diagnosis of dementia: a prospective population‐based study. J Am Geriatr Soc.

[24] Hubbard G, Cook A, Tester S, Downs M (2002). Beyond words: older people with dementia using and interpreting nonverbal behaviour. J Aging Stud.

[25] Hendrie HC (1998). Epidemiology of dementia and Alzheimer’s disease. Am J Geriatr Psychiatry.

[26] Plassman BL, Langa KM, Fisher GG, Heeringa SG, Weir DR, Ofstedal MB (2007). Prevalence of dementia in the United States: the aging, demographics, and memory study. Neuroepidemiology.

[27] Scherder E, Oosterman J, Swaab D, Herr K, Ooms M, Ribbe M (2005). Recent developments in pain in dementia. Br Med J.

[28] Bartels SJ, Horn SD, Smout RJ, Dums AR, Flaherty E, Jones JK (2003). Agitation and depression in frail nursing home elderly patients with dementia: treatment characteristics and service use. Am J Geriatr Psychiatry.

[29] Fick DM, Agostini JV, Inouye SK (2002). Delirium superimposed on dementia: a systematic review. J Am Geriatr Soc.

[30] Guthrie B, Clark SA, McCowan C (2010). The burden of psychotropic drug prescribingin people with dementia: a populationdatabase study. Age Ageing.

[31] Janzen S, Zecevic AA, Kloseck M, Orange J (2013). Managing agitation using nonpharmacological interventions for seniors with dementia. Am J Alzheimers Dis Other Demen.

[32] Van Haitsma KS, Curyto K, Abbott KM, Towsley GL, Spector A, Kleban M (2013). A randomized controlled trial for an individualized positive psychosocial intervention for the affective and behavioral symptoms of dementia in nursing home residents. J Gerontol Ser B Psychol Sci Soc Sci.

[33] Orgeta V, Qazi A, Spector AE, Orrell M (2014). Psychological treatments for depression and anxiety in dementia and mild cognitive impairment. Cochrane Database Syst Rev..

[34] Husebo BS, Ballard C, Sandvik R, Nilsen OB, Aarsland D (2011). Efficacy of treating pain to reduce behavioural disturbances in residents of nursing homes with dementia: cluster randomised clinical trial. BMJ.

[35] Etgen T, Sander D, Bickel H, Förstl H (2011). Mild cognitive impairment and dementia: the importance of modifiable risk factors. Deutsches Ärzteblatt International.

[36] Ahlskog JE, Geda YE, Graff-Radford NR, Petersen RC (2011). Physical exercise as a preventive or disease-modifying treatment of dementia and brain aging. Mayo Clinic Proceedings: 2011: Elsevier.

[37] Wang H-X, Gustafson DR, Kivipelto M, Pedersen NL, Skoog I, Windblad B (2012). Education halves the risk of dementia due to apolipoprotein ε4 allele: a collaborative study from the Swedish Brain Power initiative. Neurobiol Aging.

[38] Brooker D (2004). What is person-centred care in dementia?. Rev Clin Gerontol.

[39] Chenoweth L, King MT, Jeon Y-H, Brodaty H, Stein-Parbury J, Norman R (2009). Caring for aged dementia care resident study (CADRES) of person-centred care, dementia-care mapping, and usual care in dementia: a cluster-randomised trial. Lancet Neurol.

[40] Rokstad AMM, Røsvik J, Kirkevold Ø, Selbaek G, Saltyte Benth J, Engedal K (2013). The effect of person-centred dementia care to prevent agitation and other neuropsychiatric symptoms and enhance quality of life in nursing home patients: a 10-month randomized controlled trial. Dement Geriatr Cogn Disord.

[41] King C, Robinson A, Vickers J (2014). Online education: targeted MOOC captivates students. Nature.

[42] Lamberg JL, Person CJ, Kiely DK, Mitchell SL (2005). Decisions to hospitalize nursing home residents dying with advanced dementia. J Am Geriatr Soc.

[43] Zimmerman S, Sloane PD, Williams CS, Reed PS, Preisser JS, Eckert JK (2005). Dementia care and quality of life in assisted living and nursing homes. The Gerontologist.

[44] Volicer L (2006). Goals of care in advanced dementia: quality of life, dignity and comfort. J Nutr Health Aging.

[45] Hughes JC, Jolley D, Jordan A, Sampson EL (2007). Palliative care in dementia: issues and evidence. Adv Psychiatr Treat.

[46] Sampson E, Ritchie C, Lai R, Raven P, Blanchard M (2005). A systematic review of the scientific evidence for the efficacy of a palliative care approach in advanced dementia. Int Psychogeriatr.

[47] Mitchell SL, Kiely DK, Hamel MB, Park PS, Morris JN, Fries BE (2004). Estimating prognosis for nursing home residents with advanced dementia. J Am Med Assoc.

[48] Iliffe S, De Lepeleire J, Van Hout H, Kenny G, Lewis A, Vernooij-Dassen M (2005). Understanding obstacles to the recognition of and response to dementia in different European countries: a modified focus group approach using multinational, multi-disciplinary expert groups. Aging Ment Health.

[49] Davis DH (2004). Dementia: sociological and philosophical constructions. Soc Sci Med.

[50] Batchelor R, Bobrowicz A, Mackenzie R, Milne A (2012). Challenges of ethical and legal responsibilities when technologies’ uses and users change: social networking sites, decision-making capacity and dementia. Ethics Inf Technol.

[51] Bourkel E, Ferring D, Weber G (2012). Perceived rights of and social distance to people with Alzheimer’s disease. GeroPsych (Bern).

[52] Hertogh CM (2009). The role of advance euthanasia directives as an aid to communication and shared decision-making in dementia. J Med Ethics.

[53] Morris J (2001). Impairment and disability: constructing an ethics of care that promotes human rights. Hypatia.

